# Valve under the microscope: shining a light on emerging technologies elucidating disease mechanisms

**DOI:** 10.1136/heartjnl-2019-315236

**Published:** 2019-07-11

**Authors:** Samantha K Atkins, Elena Aikawa

**Affiliations:** 1 Center for Interdisciplinary Cardiovascular Sciences, Division of Cardiovascular Medicine, Brigham and Women’s Hospital, Harvard Medical School, Boston, MA, USA; 2 Center for Interdisciplinary Cardiovascular Sciences, Division of Cardiovascular Medicine, Brigham and Women’s Hospital, Harvard Medical School, Boston, MA, USA; 3 Center for Excellence in Vascular Biology, Division of Cardiovascular Medicine, Brigham and Women’s Hospital, Harvard Medical School, Boston, MA, USA

**Keywords:** valvular heart disease, translational cardiovascular science, vascular biology

Performing basic translational research to elucidate the underlying mechanisms driving calcific aortic valve disease (CAVD) pathogenesis is a critical step for the development of early intervention strategies. CAVD is a significant worldwide healthcare burden that is increased in ageing populations, and currently no therapies exist to delay or prevent its onset and progression. In their *Heart* paper, Gomez-Stallons *et al* histologically present the progression of calcification and associated matrix changes in tissues obtained from postmortem valves with no prior diagnosis of aortic valve stenosis (AS) and patients with AS.^1^ This is an important study that validates mineral and extracellular matrix composition changes accompanying CAVD progression.^2^ An intriguing finding was that valves without clinical AS from people over 50 years all had some form of calcification present. Clinically, only 5% of people over the age of 70 years experience CAVD that results in valvular dysfunction necessitating valve replacement. This raises a crucial unanswered question: what causes some people to develop AS while in others the disease does not progress past microcalcification? Future work that elucidates these early mechanisms will be the first step in developing targeted, precision-based medicine approaches that can identify, follow and treat high-risk patients. The findings of this research provide the initial support for more in-depth work that longitudinally studies clinical, molecular, genetic and haemodynamic properties in individuals at high risk for AS.

Future investigations should consider two important factors to help accelerate CAVD mechanistic discovery: terminology standardisation and optimised tissue utilisation. Standardised nomenclature of anatomical features and types of calcification is vital for facilitating cross-translational comparisons. Anatomically, human valve leaflets are divided into three portions: base, middle and tip.^3^ Rarely in animal studies, the base region of the leaflets will be referred to as the ‘hinge region’, but in general, this terminology should be avoided. Two types of calcification have been described: intrinsic and nodular. The latter term is well accepted for describing large calcific nodules that are grossly visible. Intrinsic calcification carries a passive connotation, misleadingly implying that valvular calcification is a degenerative process, while we now recognise that it is a highly regulated, active process.^4 5^ Therefore, microcalcifications should be described as ‘diffuse’ rather than ‘intrinsic’. The first CAVD proteome atlas recently defined leaflet regions of interest and anatomical layers.^6^ Implementation of multi-omics corroborated by histopathology helps to clearly differentiate structural and pathological features. Multi-omics could be used to address controversial issues, such as the existence of a ‘supraelastin layer’, which could be the result of aortic sclerosis that precedes calcification.

Access to a large bank of postmortem, non-diseased and surgical tissue is quite rare, and care should be taken to maximise the information gained from such precious samples. ‘Omics’ analysis on the two cohorts (postmortem vs surgical) could have led to the identification of novel proteins involved in early stages of disease. Big data analysis coupled with artificial intelligence is becoming more routine in biomedical sciences, and large ‘omics’ datasets are attractive candidates for the application of machine learning.^7^ Additionally, machine learning is well equipped to handle image analysis including histological samples. High-throughput screening of stained sections could help identify commonalities between patient subgroups stratified by various degrees of calcification. The next major step will be to ascertain the difference in subpopulations of patients with microcalcifications that do and do not develop AS, which will require longitudinal tracking that necessitates coordination between patients, clinicians and basic scientists.

A compelling finding is the apparent disparity between the histological analyses and the three-dimensional CT reconstructions. Histology detected diffuse microcalcifications at the base region, whereas CT detected a predominance of nodular calcification in the middle region. Two main factors should be considered to avoid over-interpretation of these results. First, CT was only performed on the surgical samples obtained from patients undergoing transcatheter aortic valve implantation (TAVI), and *ex vivo* CT analysis of postmortem samples was not performed. Second, diffuse microcalcifications found in the base region would be impossible to detect with CT, which suffers from low spatial sensitivity/resolution (≈200 µm). This technical limitation is most likely why only nodular calcification in the middle of the leaflet was detected. The incorporation of advanced molecular imaging tools may help address this discrepancy. ^18^F-Fluoride ligand labelled positron emission tomography (PET) coupled with CT has been shown to identify the presence of microcalcifications in atherosclerotic plaques.^8^ Application of this technology in atherosclerotic plaques demonstrated its high sensitivity: areas of microcalcifications that were CT-negative were PET-positive, most likely due to the large surface area to volume ratio of microcalcifications.^8^ While *in vivo* applications would not have been possible, *ex vivo* PET-CT in the current study would have been a potential way to track clinical progression from microcalcifications/‘diffuse’ into macrocalcifications/‘nodular’. Advancement to slow the progression of CAVD has been delayed due to our inability to identify, quantify and resolve microcalcifications and the events that lead to the initial alterations in valvular biology. Future studies should implement the *in vivo* applications of tracer technologies and advanced imaging with PET-CT.

**Figure 1:**
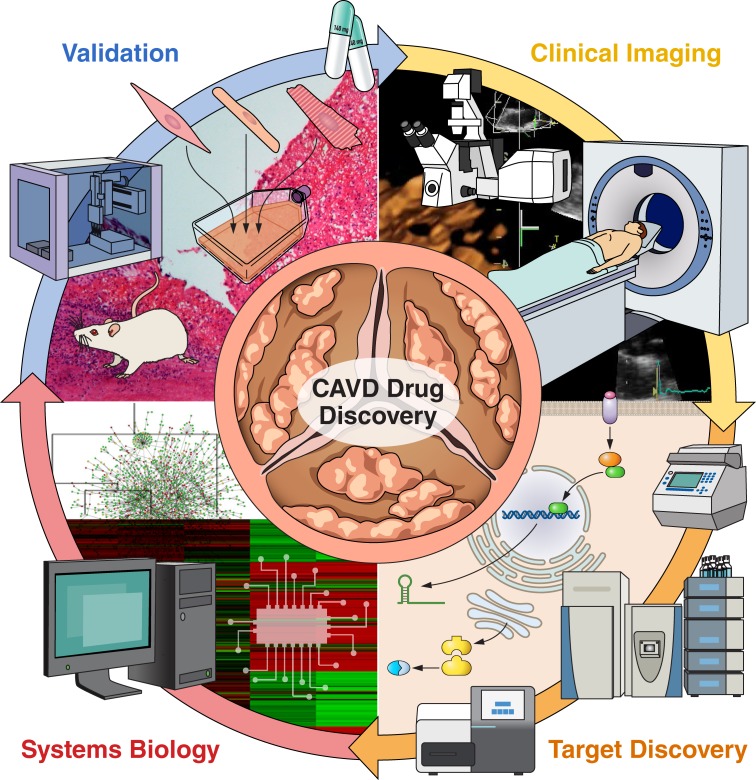
(1)Robust *in vivo* screening combined with radiotracers or near-infrared fluorescent calcium tracer to track the progression of calcific aortic valve disease in high-risk populations (hypertension, diabetes, hyperlipidaemia, bicuspid aortic valve) for early intervention and screening should be performed in parallel with (2) next-generation ‘omics’ techniques that enable collection and study of the entire genome, miRNAome, transcriptome, proteome and secretome. (3) The massive amount of data generated by this multi-omics approach is increasingly being analysed with the aid of artificial intelligence, where machine learning algorithms are employed to identify the most promising drug targets. (4) Target screening performed with *in vitro* and *in vivo* models of the pathobiology of interest is used as a first line of investigation. Care is taken to incorporate all relevant cell types, biomechanical stresses, biochemical factors, etc. These models are then utilised to carefully study the molecular mechanisms which regulate initiation and progression of disease. Once a drug/target has been validated, the cycle will continue again with follow-up patient monitoring and screening using clinical imaging.

Over the past decade, a paradigm shift has occurred in the treatment of CAVD with TAVI set to outpace surgical aortic valve replacement in the coming years.^9 10^ While patient survival and comfort are the highest priorities, an unintended consequence of this remarkable innovation is a reduction in clinical samples to perform basic CAVD research. Therefore, it is imperative that the CAVD field maximises the potential to gain tangible insights from an increasingly scarce tissue source. Robust studies will allow for the investigation of cell sources contributing to calcification, the mechanisms involved in calcification and the investigation of small molecule inhibitors to block calcification, which will aid in pharmaceutical target development with the goal of inhibiting the onset and progression of CAVD. The combination of *in vitro*, *ex vivo* and *in vivo* analyses with advanced clinical imaging and systems biology will accelerate the rate of target discovery. Systems biology and network analysis can help identify novel pathways involved in CAVD pathogenesis and reveal potential perturbagens to revert abnormal cellular phenotypes. Figure 1 shows the general schematic of the pipeline used to advance the field of CAVD research. As cardiovascular scientists, we must embrace state-of-the-art technology to advance the field forward, going beyond the microscope and into the clinic.
